# Empathy and depression among a Chinese sample: The moderating role of rumination and attentional shift

**DOI:** 10.3389/fpsyg.2022.1027298

**Published:** 2022-11-24

**Authors:** Qipeng Liu, Xiaoyun Zhao, Weidi Liu, Qianchen Liu

**Affiliations:** ^1^School of Education, Huaibei Normal University, Huaibei, China; ^2^Anhui Engineering Research Center for Intelligent Computing and Application on Cognitive Behavior, Huaibei, China

**Keywords:** attentional shift, attentional focus, rumination, depression, empathy

## Abstract

**Background:**

Although previous studies have explored the moderating role of emotional regulation strategies in the relationship between empathy and depression, no studies have studied the moderating role of attentional control in the relationship between empathy and depression. To address this research gap, the present study investigated the moderating roles of rumination and attentional control in the relationship between empathy and depression.

**Methods:**

423 participants filled out questionnaires anonymously, including Interpersonal Reactivity Index, Attention Control Scale, Self-rating Depression Scale, and Rumination Response Scale. PROCESS macro for SPSS was used for moderating effect analysis.

**Results:**

Rumination and attentional shift moderated the relationship between emotional empathy and depression. Specifically, the lower rumination or the higher attentional shift, the stronger the negative association between emotional empathy and depression. Attentional shift moderated the relationship between cognitive empathy and depression, and cognitive empathy was significantly associated with depression only among participants whose attentional shift is high.

**Conclusion:**

The study showed that rumination and attentional shift play important roles in the relationship between empathy and depression. The findings implicated that the positive role of good emotional regulation strategies and executive function for individuals in the relationship between empathy and depression.

## Introduction

Depression is a public health concern associated with serious functional impairments, including cognitive deficits, educational and learning difficulties, and increased risk of suicide (Gold et al., 2020; Chen et al., 2021). In particular, the number of depressed college students in China was gradually increasing recently (Chen et al., 2020; Li et al., 2020; Chen et al., 2022). Cognitive and emotional abnormalities were found to be the causes of depression (Tully et al., 2016; Chen et al., 2021). Previous studies have shown that executive dysfunction was an important cause of depression (Von Bastian et al., 2020; Whitmoyer et al., 2020; Yu et al., 2020). For example, depression is always accompanied by impaired attentional disengagement (Sanchez-Lopez et al., 2019; Suslow et al., 2020). The main manifestations are abnormal brain neural mechanism related to attentional disengagement (Thoma et al., 2011; Shin and Newman, 2019). Depression is also associated with abnormal emotional regulation (Powell, 2018). What is more, the abnormal brain neural mechanism of emotional regulation was also found to be a predictor of depression (Rive et al., 2013). Therefore, empathy, which includes both cognitive and emotional components, has been considered as one of the predictors of depression (e.g., Tully et al., 2016; Chen et al., 2021).

The two components of empathy may have different effects on depression (Tully et al., 2016; Chen et al., 2021), due to the fact that the two components of empathy are dissociable (Zaki and Ochsner, 2012; Goerlich-Dobre et al., 2015; Hubble et al., 2017; Noten et al., 2019; Ziermans et al., 2019; Abramson et al., 2020). The emotional component of empathy refers to the substitute emotional response to the emotional state of others with the same emotion (Emotional empathy; Pearce et al., 2017; Zaki, 2017; Toccaceli et al., 2018). The cognitive component of empathy is the understanding of the emotional state of others (Cognitive empathy; Abramson et al., 2020). Large volumes of research demonstrated that emotional and cognitive empathy are both associated with depression (Tully et al., 2016; Bennik et al., 2019; Chen et al., 2021; Zhang et al., 2021).

Existing findings on the relationship between depression and empathy was inconsistent. For example, although cognitive empathy was generally thought to be negatively associated with depression (Bennik et al., 2019), some empirical studies failed to find the asserted association (Yan et al., 2021). Similarly, some studies found the positive association of emotional empathy and depression (Bennik et al., 2019) while others found negative associations (Chen et al., 2021; Zhang et al., 2021). There were also studies demonstrated non-linear relationship between emotional and cognitive empathy and depression (Tully et al., 2016; Chen et al., 2021). The inconsistent findings collectively suggested that there may be unexplored mechanisms moderating the relationship between empathy and depression. What is more, a study using the same empathy scale and population as this paper showed that depression and empathy were significantly negatively correlated (Chen et al., 2021). Furthermore, depression and both components of empathy, including cognitive and emotional empathy, were significantly negatively correlated (Chen et al., 2021). Therefore, the results of this study should also show a significant negative association between depression and empathy, including cognitive and emotional empathy.

Cognitive empathy and emotional empathy may be linked to depression through multiple pathways. Previous studies have shown that depressed patients have poor “Theory of Mind,” which is also a form of cognitive empathy (Thoma et al., 2011). For example, people with high depression have a decreased ability to reason about the mental states of others. Therefore, people with high depression often showed lower cognitive empathy because of their poor understanding of the mental states of others (Tse and Bond, 2004). What is more, people with high depression have abnormal changes in the perspective of others and themselves, which may be related to the excessive attention of patients with high depression on information related to themselves (Thoma et al., 2011; Shin and Newman, 2019). At the same time, the abnormal activation of self-related neural networks may be an important correlate of abnormal perspective taking in people with high depression (Thoma et al., 2011). For example, people with high depression showed increased activity in the default mode network, which is a group of areas in the human brain characterized by functions of a self-referential nature, when viewing negative images (Sheline et al., 2009). As a result, people with high depression may focus too much on themselves and shift their attention too slowly when processing information about themselves and others.

Empathic concern is a component of emotional empathy, which is preferred in the assessment of one’s levels of emotional empathy (Kim and Han, 2018; Israelashvili et al., 2020;Chen et al., 2021; Zhang et al., 2021). However, empathic concern may be significantly positively correlated with depression or negatively correlated with depression (Chen et al., 2021; Zhang et al., 2021). Previous studies have suggested that the different relationship between empathic concern and depression may be due to emotional regulatory functions. Compared with those with effective emotional regulation, individuals with poor emotional regulation may be less likely to process the emotions of others, resulting in higher levels of depression (Tully et al., 2016; Powell, 2018).

Rumination moderated the relationship between emotional empathy and depression, but not the relationship between cognitive empathy and depression (e.g., Tully et al., 2016; Powell, 2018). Rumination is a repetitively negative thinking which leads individual’s attention on negative and painful thoughts (Grafton et al., 2016). Previous studies have shown that rumination had an impact not only on depression, but also on other mental disorders, such as post-traumatic stress disorder (Cox and Olatunji, 2017), sleep disorders (Nota and Coles, 2018), and eating disorders (Dondzilo et al., 2017). Rumination is not only a maladjusted emotional regulation strategy, but also a maladjusted cognitive regulation strategy. Individuals with high rumination not only focus on negative events, but also repeat them over and over again (Grafton et al., 2016). In terms of the neural mechanism, rumination involves not only the brain regions involved in emotional regulation strategies, such as amygdala (Makovac et al., 2016), but also the brain regions involved in cognitive regulation strategies, such as prefrontal cortices (Lewis et al., 2015). What is more, Tully et al. (2016) has found the moderating role of emotional regulation strategies, which mainly involve rumination, in the relationship between emotional empathy and depression, but not cognitive empathy and depression. Furthermore, the better the emotional regulation strategy, the higher the negative relationship between emotional empathy and depression may be. Moreover, rumination, as one of maladjusted emotional regulation strategies, has also been found to be closely related to depression (Joormann and Gotlib, 2010; Tully et al., 2016; Zhou et al., 2020). Therefore, the more severe rumination is, the lower the negative relationship between emotional empathy and depression.

Attentional control may also moderate the relationship between empathy and depression. Attentional control, which is a type of executive function, is critical for physical and mental health. Abnormal attentional control can lead to depression as well as increased levels of rumination (Tortella-Feliu et al., 2014; Von Bastian et al., 2020; Whitmoyer et al., 2020; Yu et al., 2020). Studies were conducted to find ways to improve people’s physical and mental health through attentional control related training (Malinowski, 2013; Dondzilo et al., 2020). Previous studies collectively demonstrated that emotional regulation strategies moderate the relationship between emotional empathy and depression (Tully et al., 2016; Powell, 2018). However, to the best of our knowledge, no study has explored whether attentional control exerts a moderating effect in the relation of emotional empathy and depression. As mentioned above, people with depression may focus too much on themselves and shift their attention too slowly when processing information about themselves and others. Therefore, the level of attentional control (including attentional shift and focus) may play an important role in the relationship between empathy and depression. Moreover, the poor attentional control will directly lead to the impaired emotional regulation strategy (Von Bastian et al., 2020; Whitmoyer et al., 2020; Yu et al., 2020), so attentional control may also moderate the relationship between emotional empathy and depression. As for the relationship between emotional empathy and depression, both attentional focus and shift may exert a moderating role. For example, emotional reappraisal is not only one of the strategies for emotional regulation, but also includes the cognitive process (Buhle et al., 2014). It was found to moderate the relationship between emotional empathy and depression (Powell, 2018). Therefore, it is reasonable to expect that attentional focus and shift moderate the relationship between emotional empathy and depression.

In contrast, in the relationship between cognitive empathy and depression, attentional shift rather than attentional focus may exert a moderating role. The moderating role of attentional control in the relationship between cognitive empathy and depression has not been studied. But impaired attentional disengagement means high levels of depression (Sanchez-Lopez et al., 2019; Suslow et al., 2020). At the same time, attentional shift rather than attentional focus was closely related to impaired attention disengagement (Taylor et al., 2016). This suggests that poor attentional shift is associated with higher levels of depression. What is more, a high level of attentional shift may also indicate a higher level of perspective-taking ability (Thoma et al., 2011), which makes people less likely to disengage from negative stimuli. Individuals are more likely to suffer from depression if they have difficulty in disengaging from negative stimuli. Therefore, in the condition of good attentional shift, cognitive empathy and depression showed a significant negative correlation. However, when the level of attentional shift is low, the individual’s depression level is generally high (Sanchez-Lopez et al., 2019; Suslow et al., 2020), which means that the negative relationship between cognitive empathy and depression become less significant.

This study seeks to examine the moderating roles of rumination and attentional control in the relationship between empathy and depression. In the current study, we propose four hypotheses. H1: there is a significant negative correlation between depression and empathy, including cognitive and emotional empathy. H2: the negative correlation between emotional empathy and depression is stronger when rumination is lower, and rumination does not moderate the relationship between cognitive empathy and depression; H3: attentional shift and focus both moderate the relationship between emotional empathy and depression; H4: attentional shift rather than attentional focus moderates the relationship between cognitive empathy and depression, and only when the level of attentional shift is high, there is a significant negative correlation between cognitive empathy and depression.

## Materials and methods

### Procedures and participants

The scales were presented in the following order: Interpersonal Reactivity Index (IRI), Attention Control Scale (ACS), Self-rating Depression Scale (SDS), and Rumination Response Scale (RRS). The participants need to answer the questions in turn. In order to screen the participants, two instructed items are also designed, requiring the participants to select a fixed option on the question. For example, please select “always” in this item (Desimone et al., 2015). If the participants did not choose the prescribed option as we asked, we will not include this questionnaire in the analysis. This process was to ensure that the conclusions are reliable.

This study collected data from college students in three cities in Eastern China: Huaibei, Xuzhou, and Nanjing. Participant was informed of the purpose of the study and the right to quit the study at any stage. Anonymity was promised to participants. The study was reviewed and approved by the Institutional Review Board of Huaibei Normal University. After removing invalid responses, 423 responses were used for this study. The mean age of the respondents was 19.29 (SD = 1.35). The sample consisted of 271 girls and 152 boys. Among the participants, 257 were from rural areas and 170 were from urban areas. Table 1 presents results from descriptive analyses.

**Table 1:** The results of descriptive statistics and correlation analysis.

	*M*	*SD*	1	2	3	4	5	6	7	8	9
1 EE	16.77	3.69	1								
2 CE	14.68	3.52	0.35^***^	1							
3 Empathy	31.44	5.93	0.83^***^	0.81^***^	1						
4 AF	24.21	3.70	0.07	0.19^***^	0.16^**^	1					
5 AS	27.96	3.97	0.09	0.29^***^	0.23^***^	0.57^***^	1				
6 AC	52.17	6.79	0.09	0.27^***^	0.22^***^	0.88^***^	0.89^***^	1			
7 Depression	39.9	7.93	−0.28^***^	−0.25^***^	−0.25^***^	−0.44^***^	−0.38^***^	−0.46^***^	1		
8 Rumination	43.53	10.5	−0.09	−0.04	−0.04	−0.34^***^	−0.17**	−0.28^***^	0.53^***^	1	
9 Age	19.29	1.35	−0.00	−0.01	−0.00	−0.06	−0.06	−0.07	0.05	0.03	1
10 Gender	1.64	0.48	0.18^***^	0.08	0.16^**^	0.03	0.01	0.02	−0.09	−0.10^*^	0.02

^*^p < 0.05, ^**^p < 0.01, and ^***^p < 0.001. EE, emotional empathy; CE, cognitive empathy; Empathy, EE + CE; AF, attentional focus; AS, attentional shift; AC, attentional control; and AC = AF + AS.

### Measures

#### Empathy

The Chinese version of IRI was used to assess emotional and cognitive empathy (Davis, 1983; Zhang et al., 2010). The IRI has been revised in China and has good reliability and validity (Zhang et al., 2010). There are 28 items in this scale. Participants were asked to rate each item on the five-point Likert scale ranging from 0 (strongly disagree) to 4 (strongly agree). The IRI contains four dimensions: perspective taking, empathic concern, fantasy, and personal distress. According to previous studies (Siu and Shek, 2005), this study measured cognitive empathy and emotional empathy with perspective taking and empathic concern, respectively. Cronbach’s α of cognitive empathy and emotional empathy in this study were 0.83 and 0.63, respectively.

#### Attentional control

Attention control was assessed by the Chinese vision of ACS (Derryberry and Reed, 2002; Yu et al., 2016). ACS contains 20 items and has two dimensions, representing two aspects of attention control ability. Participants were asked to rate each item on the four-point Likert scale ranging from 1 (never) to 4 (always). Among them, attentional focus refers to keeping attention, and attentional shift refers to transferring attention from one stimulus to another. Attentional focus includes nine items, and attentional shift includes 11 items. Cronbach’s α of attentional shift and attentional focus in this study were 0.69 and 0.73, respectively.

#### Depression

Self-rating Depression Scale was developed by Zung (1965) to measure the severity of depression. The Chinese version was revised by Duan and Sheng (2012). SDS consists of 20 declarative sentences and corresponding question items. Participants were asked to rate each item on the four-point Likert scale ranging from 1 (never) to 4 (always). SDS is a short-term self-assessment scale and questionnaire. It can effectively reflect the symptoms, severity, and changes of depression. SDS scores are not affected by age, gender, economic status, and other factors. Cronbach’s α of SDS in this study was 0.86.

#### Rumination

Rumination was assessed by the Chinese version of the RRS, which was translated and revised by Han and Yang (2009). The scale has three dimensions, namely reflective pondering, brooding, and symptom rumination (Nolen-Hoeksema, 1991). There are 22 items in RRS. Participants were asked to rate each item on the four-point Likert scale ranging from 1 (never) to 4 (always). In this study, the total Cronbach’s α of RRS is 0.93, Cronbach’s α of each sub dimension were 0.80, 0.72, and 0.91.

### Statistical analysis

SPSS 7.0 and PROCESS macro for SPSS (Hayes, 2012) was used for statistical analysis. First of all, SPSS 7.0 was used to evaluate the mean and SD of each variable and the correlation of each variable. Then, PROCESS macro for SPSS (Model No. 1; Hayes, 2012) was used to examine the moderating role of rumination and attentional control in the relationship between empathy and depression. On the premise that the moderating effect is significant, the simple slope of the variable is further analyzed. That is, the moderating variables are divided into high (higher than the average plus one standard deviation) and low (lower than the average plus one SD) groups for a simple slope test (Wu et al., 2022).

### Common method bias

There may be a risk of common method bias in collecting data by questionnaire method. Therefore, this study follows the method proposed by predecessors to control common method bias (anonymous method and reverse scoring of some items), and uses Harman single factor test to test common method bias (Podsakoff et al., 2003). There are 17 factors with characteristic root greater than 1. The first factor explains 19.26% of the total variation, which is less than the critical value of 40%, which confirms that there is no serious common method deviation in this study.

## Results

### Descriptive statistics and correlation analysis

The results of descriptive statistics and correlation analysis are shown in Table 1.

### Rumination and attentional shift moderate the relationship between emotional empathy and depression

In this study, SPSS macro program (Hayes, 2012) was used for data processing. Before formal data processing, all variables should be standardized. Furthermore, the moderated effects of rumination, attentional shift, and attention focus on emotional empathy and depression were tested. The research results are shown in Table 2. The results showed that rumination and attentional shift had significant moderated effects on the relationship between emotional empathy and depression. Through simple slope analysis, we further investigated the moderated effect of rumination and attentional shift on the relationship between emotional empathy and depression.

**Table 2:** Results of moderating analysis on the effects of depression.

Predictors	*R*	*R* ^2^	*F*	*β*	Bootstrap down	Bootstrap up	*t*
Moderator: rumination							
EE	0.58	0.34	71.87^***^	−0.53	−0.68	−0.34	−5.97^***^
Rumination				0.39	0.33	0.45	12.83^***^
EE × Rumination				0.02	0.00	0.03	2.20^*^
CE	0.57	0.33	69.07^***^	−0.52	−0.70	−0.34	−5.69^***^
Rumination				0.39	0.33	0.45	12.95^***^
CE × Rumination				0.00	−0.01	0.02	0.35
Moderator: AF							
EE	0.50	0.26	47.74^***^	−0.53	−0.71	−0.35	−5.78^***^
AF				−0.90	−1.08	−0.72	−9.90^***^
EE × AF				−0.03	−0.07	0.01	−1.36
CE	0.486	0.219	39.18^***^	−0.39	−0.59	−0.20	−3.94^***^
AF				−0.86	−1.05	−0.67	−9.01^***^
CE × AF				−0.01	−0.05	0.04	−0.34
Moderator: AS							
EE	0.46	0.21	49.92^***^	−0.52	−0.70	−0.33	−5.52^***^
AS				−0.69	−0.86	−0.51	−7.77^***^
EE × AS				−0.05	−0.09	0.00	−1.98^*^
CE	0.42	0.18	30.22^***^	−0.36	−0.57	−0.16	−3.47^***^
AS				−0.60	−0.79	−0.41	−6.24^***^
CE × AS				−0.07	−0.12	−0.02	−2.61^**^

*p < 0.05, **p < 0.01, and ***p < 0.001, EE, emotional empathy; CE, cognitive empathy; AS, attentional shift; and AF, attentional focus.

**Figure 1:**
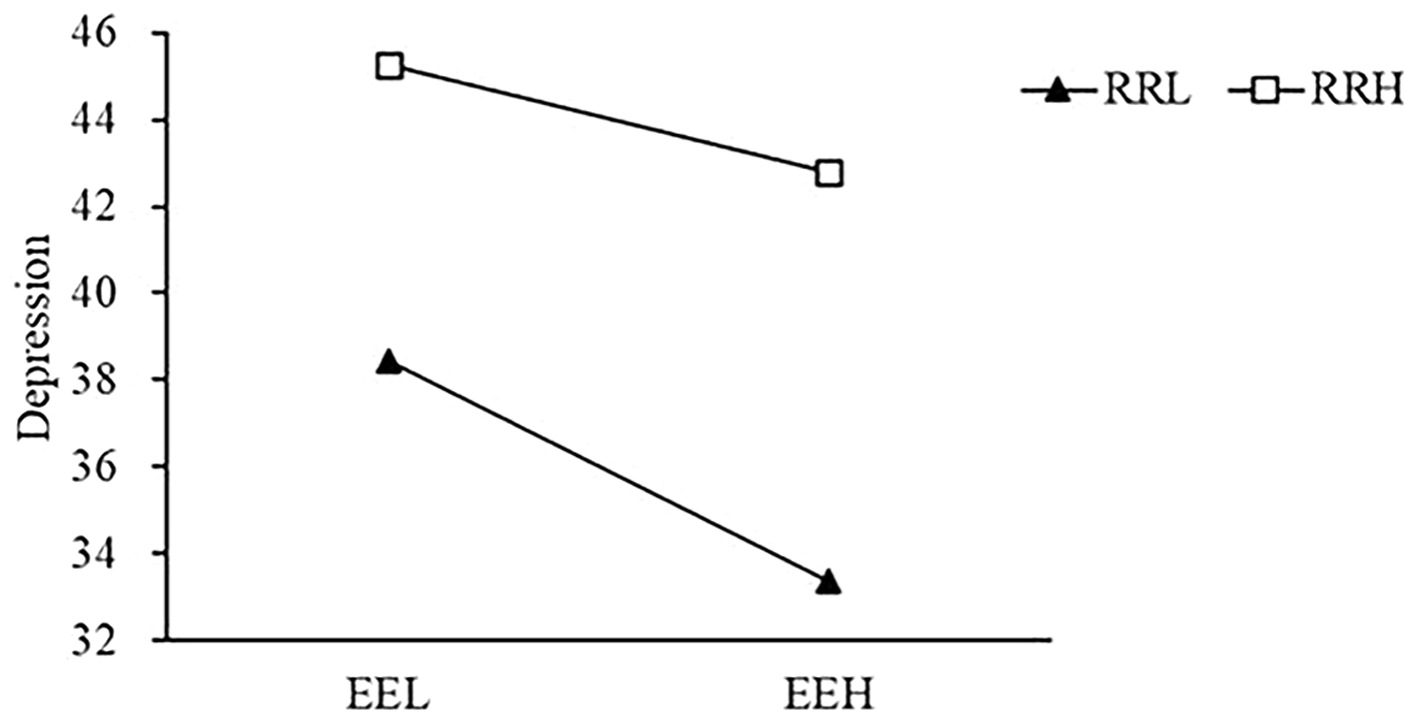
Moderated effect of rumination on the relationship between emotional empathy and depression. RRL, low rumination; RRH, high rumination; EEL, low emotional empathy; and EEH, high emotional empathy.

**Figure 2:**
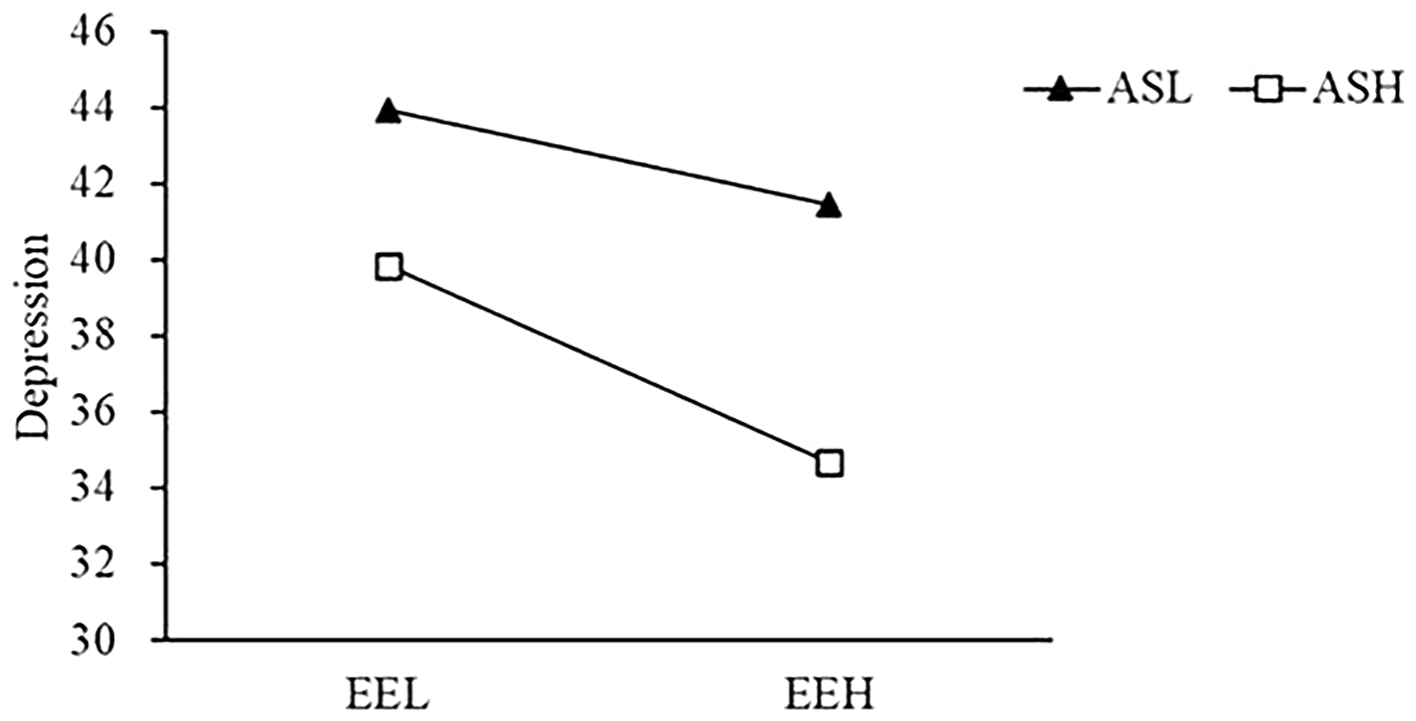
Moderated effect of attentional shift on the relationship between emotional empathy and depression. ASL, low attentional shift; RRH, high attentional shift; EEL, low emotional empathy; and EEH, high emotional empathy.

The high rumination/attentional shift group (higher than the average plus one standard deviation) and the low rumination/attentional shift group (lower than the average plus one standard deviation) were selected for simple slope test (Wu et al., 2022). Low rumination group: Simple slope = −0.71, 95% confidence interval was [−0.93, −0.45], *p* < 0.001. High rumination group: Simple lope = −0.35, and the 95% confidence interval was [−0.56, −0.11], *p* < 0.01 (see Figure 1). Low attentional shift group: Simple slope = −0.34, 95% confidence interval was [−0.60, −0.07], *p* < 0.05. High attentional shift group: Simple slope = −0.70, 95% confidence interval was [−0.95, −0.45], *p* < 0.001 (see Figure 2). In addition, attentional focus did not play a moderated role in emotional empathy and depression.

### Attentional shift moderated the relationship between cognitive empathy and depression

**Figure 3:**
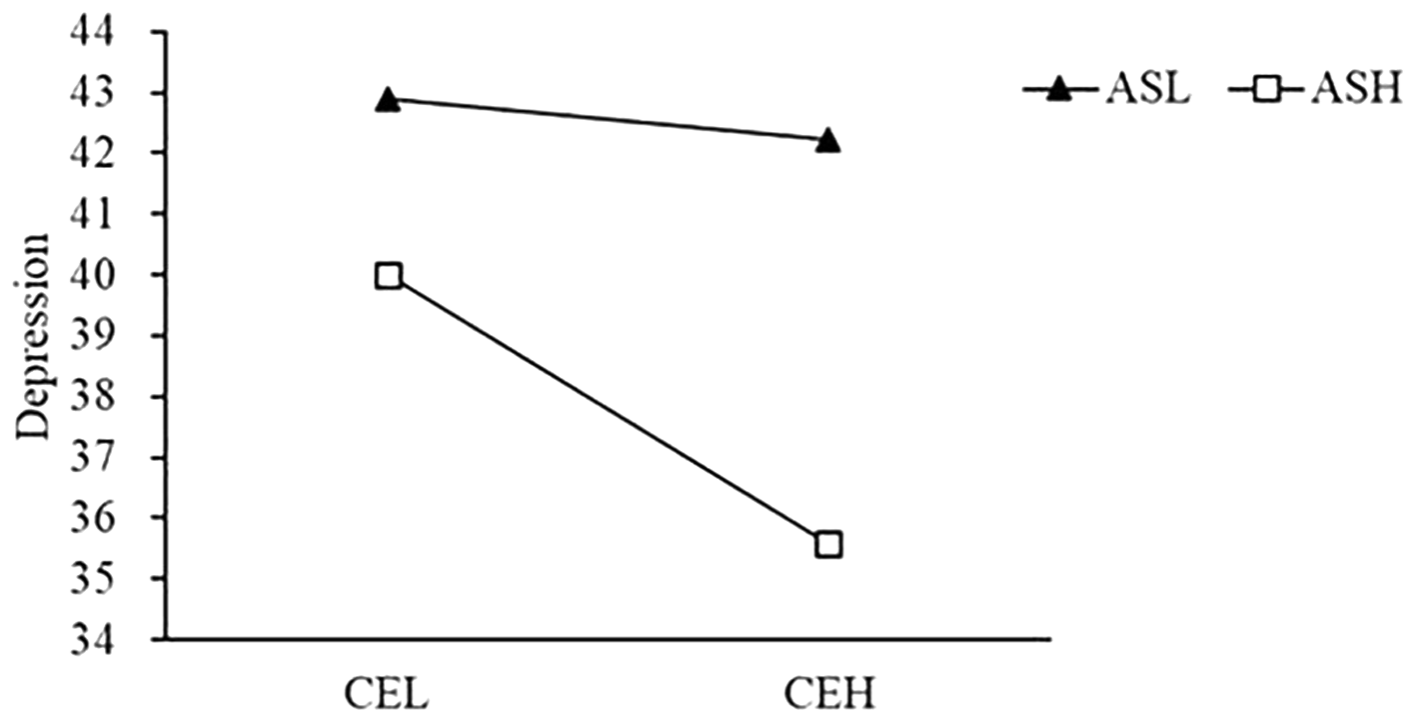
The moderating effect of attentional shift on the relationship between cognitive empathy and depression. ASL, low attentional shift; ASH, high attentional shift; CEL, low cognitive empathy; and CEH, high cognitive empathy.

The moderated analysis of attention control and rumination in the relationship between cognitive empathy and depression showed that attentional shift had a significant moderated effect on cognitive empathy and depression, while attentional focus and rumination did not play a moderated role in the relationship between cognitive empathy and depression. The results are shown in Table 2. Through simple slope analysis, we further investigated the moderated effect of the relationship between cognitive empathy and depression. Low attentional shift group: Simple lope = −0.09, 95% confidence interval was [−0.37, 0.18], *p* > 0.05; High attentional shift group: Simple lope = −0.63 and 95% confidence interval was [−0.93, −0.33], *p* < 0.001 (See Figure 3).

## Discussion

This study investigated the moderating role of rumination and attentional control in the relationship between empathy and depression. The results showed that there was a significant negative correlation between depression and empathy, which supported H1. Rumination moderated the relationship between emotional empathy and depression, but not cognitive empathy and depression. Moreover, attentional shift rather than attentional focus moderated the relationship between emotional empathy and depression, as well as the relationship between cognitive empathy and depression. This further pointed to the relevance of rumination and attentional control in the relationship between empathy and depression.

### Moderating role of rumination in the relationship between emotional empathy and depression

Supporting H2, our results showed that rumination moderated the relationship between emotional empathy and depression, but not cognitive empathy and depression. Our results are consistent with previous research in other cultural contexts (Tully et al., 2016; Powell, 2018). Although the level of depression decreased significantly with the increase of emotional empathy in both high and low groups, compared with the high rumination group, the low rumination group has a greater reduction. According to emotional regulation theory (Tully et al., 2016; Gold et al., 2020; Chen et al., 2021), the impairment emotional regulation is closely related to depression. Compared with low rumination, emotional empathy and rumination may have a stronger relationship in high rumination, which weakens the negative correlation between emotional empathy and depression to some extent (Thompson et al., 2022). Therefore, the negative correlation between emotional empathy and depression was stronger when rumination was lower.

Higher level of rumination was found to be associated with more negative emotions (Grafton et al., 2016). In this case, individuals with low rumination may be less likely to be caught up in the negative emotions of others than individuals with high rumination, thus reinforcing the negative relationship between emotional empathy and depression. Rumination is an automated form of thinking that allows individuals to continually recall negative events (Grafton et al., 2016). This way of thinking makes it difficult for individuals to disengage from negative emotions, which further attenuates the negative association between emotional empathy and depression. Therefore, rumination may moderate the relationship between emotional empathy and depression through emotional regulation and cognitive regulation.

### Moderating role of attentional shift in the relationship between emotional empathy and depression

Our findings showed that only attentional shift moderated the relationship between emotional empathy and depression, which contradicted H3. There are two plausible explanations. First, there may be some overlap between attentional shift and emotional regulation strategies. Powell (2018) found that cognitive reappraisal can moderate the relationship between emotional empathy and depression, and cognitive reappraisal involves the transformation of perspective taking, that is, it is necessary to consider problems from different aspects rather than focusing on one aspect (Buhle et al., 2014). Therefore, attentional shift can moderate the relationship between emotional empathy and depression. Second, attentional focus may not be involved in the emotional and cognitive regulation related to emotional empathy, which will moderate the relationship between emotional empathy and depression. Therefore, attentional focus may not moderate the relationship between emotional empathy and depression.

This result reflected the process by which the negative relationship between emotional empathy and depression diminishes if individuals are unable to disengage from negative emotions. Therefore, if individuals have a low level of attentional shift, it is likely to weaken the relationship between emotional empathy and depression. At the same time, although attentional focus was significantly negative associated with rumination, it did not appear to be involved in the emotional and cognitive regulation related to emotional empathy. These results further suggested that it may be only the emotional and cognitive regulation related to attentional shift that are important factors in the relationship between emotional empathy and depression.

### Attentional shift not focus moderated the relationship between cognitive empathy and depression

In terms of executive function related to depression, we found attentional shift rather than attentional focus moderated the relationship between cognitive empathy and depression. This is consistent with H4. Specifically, when the level of attentional shift was low, the relationship between cognitive empathy and depression was no longer significant. When the level of individual attentional shift was high, the negative correlation between cognitive empathy and depression is significant, which is also consistent with previous research results, that is, in some studies, cognitive empathy and depression showed significantly negative correlation (Bennik et al., 2019) or not significantly correlated (Yan et al., 2021).

When the level of attentional shift was high, cognitive empathy and depression showed significantly negative correlation. This may be because, individuals, with good attentional shift, will not have difficulty in attentional shift from negative stimuli, so they will not focus on negative emotions when they feel the negative emotions of others. Therefore, when attentional shift is good, individuals with higher cognitive empathy can understand the emotions of others and are less susceptible to the influence of the emotions of others.

However, when the level of attentional shift was low, the negative relationship between cognitive empathy and depression was no longer significant. Previous studies have shown that when individuals had difficulty in attentional shift, their level of depression was generally high (Sperduti et al., 2017; Sanchez-Lopez et al., 2019; Suslow et al., 2020; von Bastian et al., 2020; Whitmoyer et al., 2020; Yu et al., 2020). Therefore, when the level of attentional shift was low, regardless of the level of cognitive empathy, the depression level of individuals was generally high, which also leaded to the negative correlation between cognitive empathy and depression was no longer significant. What is more, when the level of attentional shift was low, the perspective-taking ability in cognitive empathy was poor. At this time, even though individuals can understand the emotions of others, it was difficult to consider things from the perspective of others (Tusche et al., 2016; Ferguson and Cane, 2017; Goodhew and Edwards, 2022). Therefore, the perspective-taking ability of cognitive empathy may play a key role in the relationship between depression and cognitive empathy.

### Limitations

This study is not without limitation. First, this study is a cross-sectional study, and thus causal relationship between variables cannot be claimed. Second, future researchers need to consider using different measures of empathy to test the moderating roles of rumination and attentional control in the relationship between empathy and depression. For example, this study used IRI to measure empathy. The results may be different if we sued questionnaire of cognitive and emotional empathy (QCAE; Goodhew and Edwards, 2021, 2022). Third, this study did not include personal distress in the analysis. The relationship between rumination and attentional control in personal distress and depression needs to be further explored in the future.

## Conclusion

In conclusion, this study found the significantly negative relationship between empathy and depression. In terms of emotional regulation strategies, rumination moderated the relationship between emotional empathy and depression. In terms of executive function, attentional shift not only moderated the relationship between cognitive empathy and depression, but also moderated the relationship between emotional empathy and depression, which further advanced the understanding of the relationship between empathy and depression. The findings implicated that the positive role of good emotional regulation strategies and executive function for individuals in the relationship between empathy and depression.

## Data availability statement

The original contributions presented in the study are included in the article/Supplementary material, further inquiries can be directed to the corresponding author.

## Ethics statement

The studies involving human participants were reviewed and approved by the Ethics Committee of Huaibei Normal University. The patients/participants provided their written informed consent to participate in this study.

## Author contributions

QipL, XZ, and QiaL contributed to design of the study, collected the database, and performed the statistical analysis. QipL wrote the first draft of the manuscript. QipL and WL polished the manuscript. All authors contributed to the article and approved the submitted version.

## Conflict of interest

The authors declare that the research was conducted in the absence of any commercial or financial relationships that could be construed as a potential conflict of interest.

## Publisher’s note

All claims expressed in this article are solely those of the authors and do not necessarily represent those of their affiliated organizations, or those of the publisher, the editors and the reviewers. Any product that may be evaluated in this article, or claim that may be made by its manufacturer, is not guaranteed or endorsed by the publisher.

## Supplementary material

The Supplementary material for this article can be found online at: https://www.frontiersin.org/articles/10.3389/fpsyg.2022.1027298/full#supplementary-material
